# Continuity of active commuting to school across two generations: the Cardiovascular Risk in Young Finns Study

**DOI:** 10.1093/eurpub/ckaf084

**Published:** 2025-06-03

**Authors:** Tuuli H Suominen, Tuomas Kukko, Xiaolin Yang, Katja Pahkala, Suvi Rovio, Mirja Hirvensalo, Mika Kähönen, Olli Raitakari, Tuija H Tammelin, Kasper Salin

**Affiliations:** Faculty of Sport and Health Sciences, University of Jyväskylä, Jyväskylä, Finland; Likes, School of Health and Social Studies, Jamk University of Applied Sciences, Jyväskylä, Finland; Likes, School of Health and Social Studies, Jamk University of Applied Sciences, Jyväskylä, Finland; Likes, School of Health and Social Studies, Jamk University of Applied Sciences, Jyväskylä, Finland; Research Centre of Applied and Preventive Cardiovascular Medicine, University of Turku, Turku, Finland; Centre for Population Health Research, University of Turku and Turku University Hospital, Turku, Finland; Paavo Nurmi Centre and Unit for Health and Physical Activity, University of Turku, Turku, Finland; Research Centre of Applied and Preventive Cardiovascular Medicine, University of Turku, Turku, Finland; Centre for Population Health Research, University of Turku and Turku University Hospital, Turku, Finland; Department of Public Health, University of Turku and Turku University Hospital, Turku, Finland; Faculty of Sport and Health Sciences, University of Jyväskylä, Jyväskylä, Finland; Department of Clinical Physiology, Faculty of Medicine and Health Technology, Tampere University, Tampere, Finland; Department of Clinical Physiology, Tampere University Hospital, Tampere, Finland; Research Centre of Applied and Preventive Cardiovascular Medicine, University of Turku, Turku, Finland; Centre for Population Health Research, University of Turku and Turku University Hospital, Turku, Finland; Department of Clinical Physiology and Nuclear Medicine, Turku University Hospital, Turku, Finland; Likes, School of Health and Social Studies, Jamk University of Applied Sciences, Jyväskylä, Finland; Faculty of Sport and Health Sciences, University of Jyväskylä, Jyväskylä, Finland

## Abstract

Active commuting to school (ACS) may markedly contribute to overall physical activity (PA) among youth, but ACS levels have declined in recent decades. Parents significantly influence their children’s PA and commuting behaviours, and lifestyle habits are often transferred from parents to offspring. This study investigated whether parents’ ACS during their youth was associated with their offspring’s ACS at similar ages. In this study, 660 parent–offspring pairs self-reported their mode of school commuting: parents during 1980–86 (generation G1, ages 9–18, 53% female) and offspring in 2018 (generation G2, ages 7–20, 52% female). A path model was constructed to examine the association of ACS in G1 with ACS in G2, adjusted for generation-specific covariates (distance to school, school grade, gender, living area, parental education, and family income). Standardized path coefficients are reported, concentrating on their direction and relative strength. Distance to school was inversely associated with ACS in both generations (*β* ≤ −0.75; SE = 0.03; *P* < .001). Family income was directly associated with ACS in G1 (*β* =  0.18; SE = 0.05; *P* < .01). ACS in G1 was directly associated with ACS in G2 (*β* =  0.14; SE = 0.05;*P* < .01). A positive, albeit modest, link was found between parents’ ACS during their youth and their offspring’s ACS at similar ages, after adjusting for multiple important covariates. This intergenerational link could inform public health initiatives to foster sustainable and healthy commuting behaviours that benefit current and future generations. Ensuring accessible distances to school remains important.

## Introduction

Active commuting to school (ACS), such as walking or cycling, is a potential source of daily physical activity (PA) for children and adolescents. ACS provides numerous health and environmental benefits [1–3] and is recognized as a key strategy to address the global challenge of insufficient PA among youth [4, 5]. It is a low-cost and accessible way to integrate PA into daily life, even for those not involved in sports or other leisure activities. Despite these advantages, ACS has declined globally over recent decades [6–8], raising concerns about the long-term health implications for younger generations. Identifying the contributors and driving forces of ACS is essential for reversing this trend and reducing physical inactivity.

Lifestyle habits, including PA, are often transferred from parents to their offspring. During childhood, parents are frequently identified as key influencers in their children’s PA [9–11]. Particularly for younger children, parents are the primary decision-makers regarding their children’s commuting modes. Their personal experiences, attitudes, and beliefs about ACS can significantly shape their children’s commuting behaviours [12–14]. The concept of intergenerational transmission refers to the transfer of abilities, behaviours, characteristics, and values from parents to offspring [15, 16], influenced by a complex interplay of genetic and environmental factors [17]. This transmission can occur through multiple pathways, including modelling, imitation, and socialization within the family [18–20]. It may be sex-specific and dependent on the child’s age, with stronger effects observed in younger children, and influenced by the family’s socioeconomic status [15]. The continuity of ACS across generations has been sparsely studied.

ACS is influenced by various sociodemographic, environmental (e.g. distance to school, infrastructure, and safety), social, and policy-related factors [8, 21], which may vary across countries and cultures. We and others have previously reported that higher levels of parental PA, including active commuting behaviours, are associated with higher ACS levels among their children [22–28]. Additionally, a study by Ziviani *et al.* [14] found that children were more likely to commute actively to school if their parents had done so. However, no prospective studies have been conducted on the impacts of parents’ own childhood and adolescent school commuting habits on their children’s commuting behaviours at similar ages.

The purpose of this prospective two-generation study, conducted in Finland, was to examine whether parents’ ACS during their childhood and adolescence (between ages 9 and 18) is associated with their offspring’s ACS at similar ages. Through this approach, we aim to expand the knowledge of the driving forces and contributors to active commuting behaviours and the development and maintenance of a physically active lifestyle in high-income areas. Several important covariates of ACS were considered for both generations, including age, gender, and family SES, which could also influence the intergenerational transmission of ACS. Additional analyses were conducted for different school grades (primary, lower secondary, and upper secondary school) due to the potential decline in parental influence on children’s PA with increasing age.

## Methods

### Study design and participants

This study is part of the ongoing, prospective, population-based Cardiovascular Risk in Young Finns Study (YFS) [29, 30], undertaken on six age cohorts born in 1962, 1965, 1968, 1971, 1974, and 1977. At the baseline in 1980, the study included 3596 children and adolescents aged 3–18 years (83.2% of those invited, 51% females). These participants were randomly selected from the catchment areas of five Finnish university hospitals. Follow-up studies have been conducted in 3-to-9-year intervals. During the latest follow-up in 2018–20, the children of the original YFS participants were also invited to participate [29]. A total of 5696 offspring (aged 3–37) were invited, of whom 2762 (48.5%) attended the study visit. The study was approved by the ethical committees of each of the five universities, and a written informed consent was obtained from all participants in accordance with the Helsinki Declaration.

This study utilizes data from the original YFS participants (Generation 1, G1) collected in the years 1980, 1983, and 1986 (at ages 9, 12, 15, and 18), and from their 7-to-20-year-old offspring (Generation 2, G2) collected in 2018. G1 participants aged 6 and 21 were excluded as they were not in compulsory education in Finland and thus were not commuting to school. Among 18–20-year-olds, only those still participating in schoolwork (e.g. vocational education or upper secondary school) were included. The data of G1 participants (the parents) were matched with the G2 participants (the offspring) by school grade and age (Supplementary Table S1). In total, 660 parent–child pairs with valid data on covariates of ACS were available, including 381 parent–child pairs of primary school-aged children, 155 pairs of lower secondary school-aged children, and 124 pairs of upper secondary school-aged children.

### Measurements

#### Commuting to school

The mode of commuting to school was assessed using a questionnaire with similar questions for both generations. The commuting modes were coded as follows: 1 = own car or carpool, 2 = public transport, 3 = walking, 4 = cycling. These were further categorized into ‘passive’ (car or public transport) and ‘active’ (walking or cycling) modes. Separate questions were applied for summer and winter conditions, except at baseline in 1980. The 1980 questionnaire was administered in the fall (for the vast majority of respondents in October) when the weather conditions were comparable to those experienced during summer (as opposed to cold and snowy conditions typical of winter). Moreover, the commuting modes reported in 1980 were found to align with summer commuting patterns observed at other follow-up points. Consequently, the 1980 data were used as part of the summer commuting data, and only summer commuting is reported in this article.

#### Covariates

Similar questions were applied to both generations to assess participants’ age, gender, distance to school, and parental educational attainment level and family income, which were used as indicators of family SES. The school grade within the Finnish educational system was assigned based on the participant’s age: primary school for ages 7–12, lower secondary for ages 13–15, and upper secondary for ages 16–20 years. Distance to school was reported in kilometres, rounded to the nearest 100 metres, and truncated at 10 km. Parental education was categorized as low (comprehensive school: primary and lower secondary school), middle (upper secondary: high school/vocational school), and high (polytechnic and university levels). For G1 participants, parental education was queried from both mothers and fathers, and a higher value was used if data from both parents were available. For G2 participants, parental data were utilized from the G1 participant within the parent–child pairs, either mother or father. Family income was divided into low, middle, and high tertiles based on the value of money over time and the response categories in the questionnaire. The living area in the G1 questionnaire included four response options (1 = city centre, 2 = suburb, 3 = rural community, 4 = dispersed settlement area), which were recategorized as urban (1 and 2) and rural (3 and 4) areas. For G2 participants, corresponding categories were derived from postal codes and the Finnish Environment Institute’s GIS-based classifications of neighbourhood urbanity [31].

#### Statistical analysis

**Figure 1.**
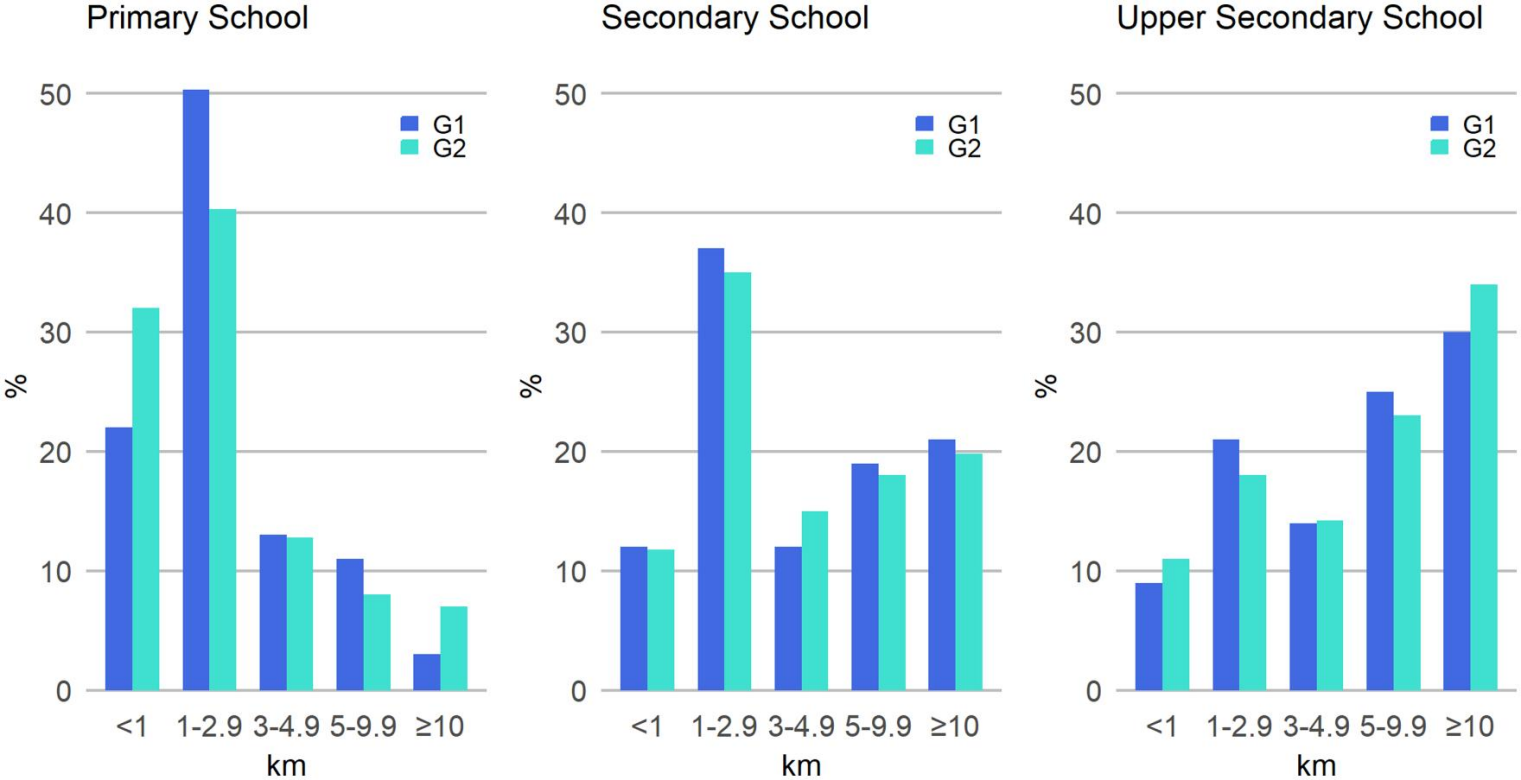
Distributions of home–school distances among G1 and G2 generations according to school grade (primary school *n* = 381, lower secondary school *n* = 155, and upper secondary school *n* = 124, for both G1 and G2).

Four path models were fitted to investigate the intergenerational association of ACS. One model was fitted for the full dataset, and three subsequent models were fitted for three school grades: primary school, lower secondary school, and upper secondary school. A path model is a special case of structural equation models that deals with complex causal connections and other associations with observed variables only. Each path model had two dichotomized outcomes: ACS in G1 and G2. The outcomes were regressed by the generation-specific covariates: distance to school (truncated at 10 km), family income, parental education, gender, living area, and, in the model of the full dataset, the participants’ school grade. Additionally, the G2 participants’ commuting mode was further regressed by the school commuting mode of the parent (G1) (Fig. 1). All the models were adjusted for the time lag between G1 and G2 observations. Categorized covariates were assumed to be ordinal and linearly associated with the outcome variables. Family structure was accounted for using a multilevel design in the path analysis. The family ID variable was used to define the correlated data clusters. Path models were fitted in Mplus by Monte Carlo integration using the probit link function. Standardized path coefficients and their standard errors are reported and interpreted, focusing on the sign and relative strength. Finally, the main results, statistically significant associations for ACS in G2, were interpreted on a probability scale. Probabilities were estimated based on the raw parameter estimates of the path model and inverse probit transformations.

Models were evaluated with a chi-squared test (acceptable fit *P* > .05), comparative fit index (CFI, acceptable fit ≥ 0.90), Tucker–Lewis Index (TLI, acceptable fit ≥ 0.90), Root Mean Square Error of Approximation (RMSEA, acceptable fit ≤ 0.08), and Standardized Root Mean Square Residuals (SRMR, acceptable fit ≤ 0.08) [32–34]. Analyses were conducted with R 4.2.1 and MPLUS 8.8. The significance was set at 5% in all analyses.

#### Missing data

For G1, missing data on living area, parental education, and family income were supplemented with corresponding data from previous follow-ups. For G2, a case-wise deletion approach was applied for missing covariate data. Missingness within the outcome variables was assumed to be missing at random, and the missing outcomes were multiply imputed within the analysis. The proportion of completely observed outcomes is 99.5%.

## Results

Commuting modes and covariates of ACS among G1 and G2 are shown in Table 1. In both generations, slightly over half of the participants were female. More than half of the participants were of primary school age, whereas approximately one-fourth were of lower secondary school age and one-fifth were of upper secondary school age. Less than half of the G1 participants lived in urban areas, whereas the proportion among G2 was over two-thirds. Parents were less educated in the G1 generation than in the G2 generation. ACS was more common among G1 than G2.

**Table 1.** Characteristics of participants according to generation

	G1 generation, parents (born 1962–71)	G2 generation, offspring (born 1998–2011)
Gender, *n* (%)		
Female	351 (53.2)	342 (51.8)
Male	309 (46.8)	318 (48.2)
Age, years [mean, standard deviation (SD)]	12.7 (3.5)	12.3 (3.4)
School grade, %		
Primary (7–12-year-olds)	57.7	57.7
Lower secondary (13–15-year-olds)	23.5	23.5
Upper secondary (16–20-year-olds)	18.8	18.8
Distance to school, km (mean, SD)^a^	3.3 (3.2)	3.5 (3.3)
Living area^b^, %		
Rural	52.9	30.6
Urban	47.1	69.4
Parental education^b^, %		
Elementary	48.8	4.4
Secondary	36.2	36.8
Academic	15.0	58.8
Family income^b^, %		
Low	20.8	21.2
Middle	35.5	39.1
High	43.8	39.7
Commuting mode, %		
Passive	31.1	36.5
Active	68.9	63.5

*Note*: Values are percentages unless otherwise noted.

a Truncated at 10.0 km;

b Missing data are supplemented with data from previous follow-ups.

**Figure 2.**
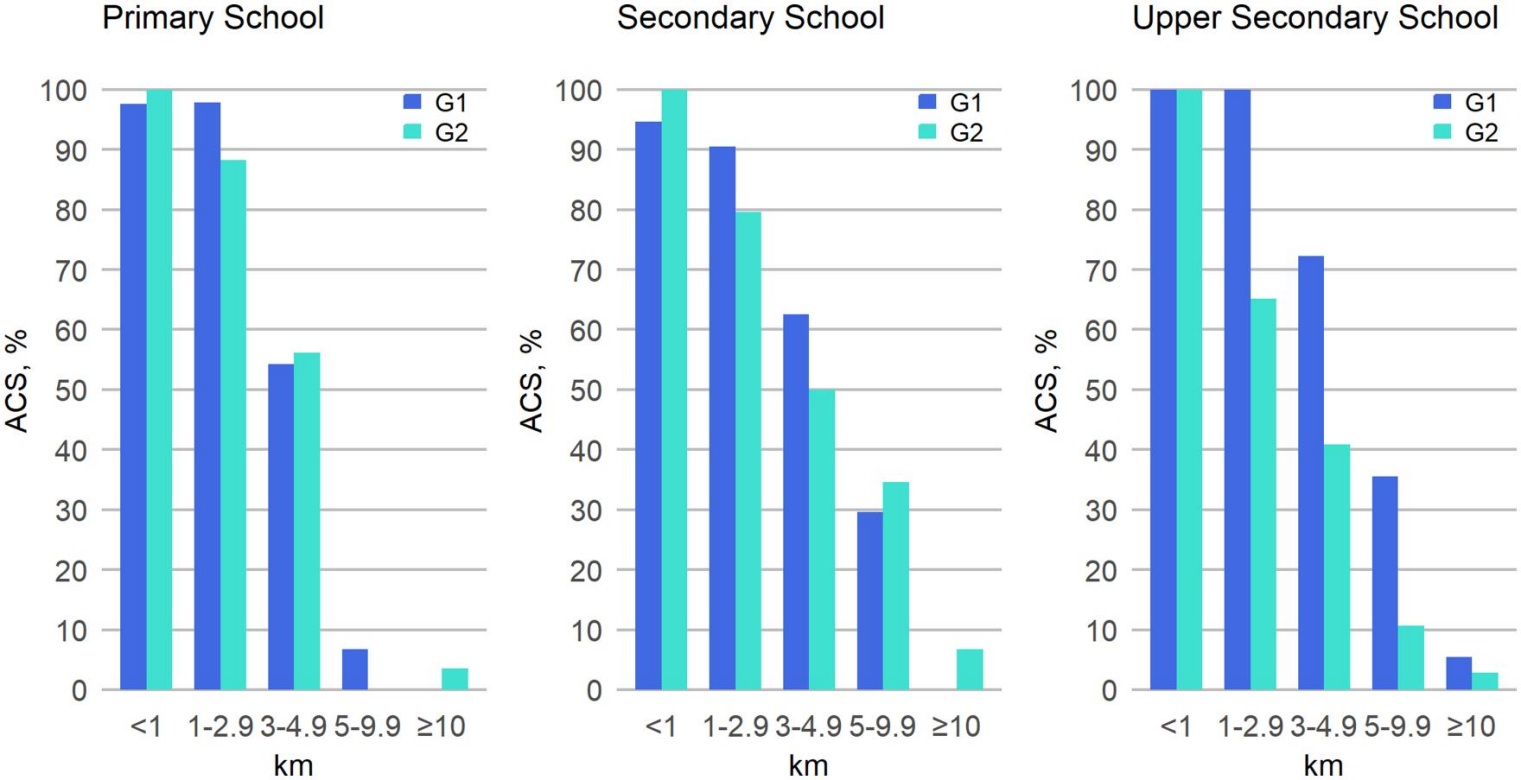
Unadjusted proportions of active commuters among G1 and G2 generations according to distance to school, separately for the school grades (primary school *n* = 381, lower secondary school *n* = 155, and upper secondary school *n* = 124, for both G1 and G2).

Descriptive characteristics across different school grades are summarized in Supplementary Table S2 and Figs 1 and 2. Figure 1 illustrates the distributions of home–school distances for the G1 and G2 generations across different school grades. In both generations, slightly over 70% of participants of primary school age had a home–school distance of less than 3 km. For lower and upper secondary students, the corresponding proportions were approximately 50% and 30%, respectively. Among primary school students, the proportions of home–school distances decreased consistently at distances of 3 km and beyond. Conversely, for lower and upper secondary students, the opposite phenomenon was observed. The largest intergenerational differences in the proportions of home–school distances were observed in primary school at distances less than 3 km. Specifically, 22% of G1 and 32% of G2 participants at primary school had a home-school distance of less than 1 km, whereas the proportions of those with a home–school distance of 1–2.9 km were 50% for G1 and 40% for G2.

Figure 2 displays the unadjusted proportions of active commuters among G1 and G2 generations according to distance to school, separated by school grades. Among primary school-aged participants, 88%–100% commuted actively to school if the distance was less than 3 km. For distances of 3–5 km, only slightly over half of the primary school students commuted actively. At lower and upper secondary school grades, the proportions of ACS declined relatively steadily with increased distances. G2 participants at lower and upper secondary school grades had lower proportions of ACS compared to G1 across most distance classes.

**Figure 3.**
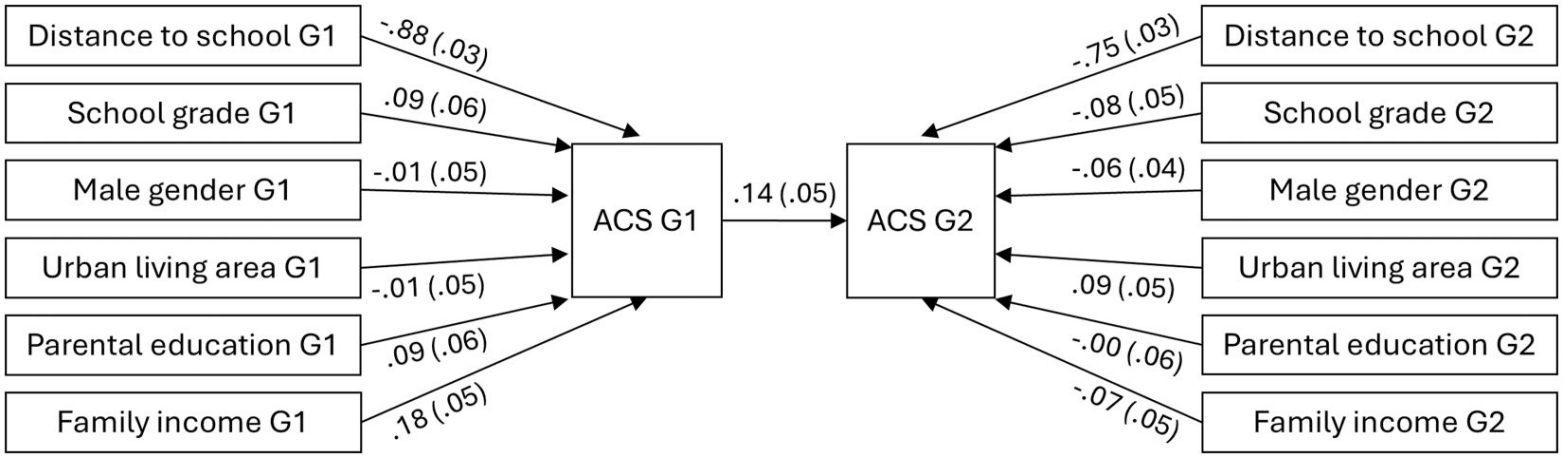
Directed acyclic graph of the path model (*n* = 660, for both G1 and G2) of ACS across two generations with its covariates. Values are standardized regression coefficients (SE). Model fit: X^2^ = 9.9 (df 11); *P* = .538; CFI = 1; TLI = 1; RMSEA = 0; and SRMR = 0.066.

The path model of ACS across two generations is shown in Fig. 3. Distance to school was inversely associated with ACS in both generations [*β* ≤ −0.75; standard error (SE) = 0.03; *P* < .001]. Among G1, higher family income was positively associated with ACS (*β*  =  0.18; SE = 0.05; *P* = .001). ACS among G1 was positively associated with ACS among G2 (*β*  =  0.14; SE = 0.05; *P* = .002). Model fit evaluations showed adequate fit (Fig. 3).

The effect sizes of home-school distance and parental ACS for G2 are illustrated on a probability scale in Supplementary Table S3. Fixing all other covariates to their mean (or mode) levels, an increase in home–school distance from 3.0–4.0 km decreased the estimated probability of ACS by 13 percentage points (odds ratio 0.46). Correspondingly, parental ACS increased the estimated probability of ACS in the G2 generation by 4 percentage points (odds ratio 1.28).

Path models for ACS within primary, lower secondary, and upper secondary school grades are shown in Supplementary Fig. S4. Distance to school was inversely associated with ACS in both generations across all school grades (*β* ≤ −0.75; SE ≤ 0.08; *P* < .001). For primary school-aged participants, family income was positively associated with ACS among G1 (*β*  =  0.26; SE = 0.08; *P* = .001), whereas among lower secondary school students, family income was inversely associated with ACS among G2 (*β* = −0.22; SE = 0.10; *P* = .024). ACS in G1 was positively associated with ACS in G2 only for primary school-aged participants (*β*  =  0.29; SE = 0.09; *P* = .001). Model fit evaluations showed, in part, inadequate fit for the models (Supplementary Fig. S4).

## Discussion

This prospective two-generation study among Finnish youth examined ACS across two cohorts: parents (G1), who attended school during the 1980s, and their offspring (G2), who attended school during the late 2010s. With nearly 700 parent–offspring pairs, we observed that parents’ ACS during their youth was positively, albeit modestly, linked with their children’s ACS after adjusting for several key covariates in both generations. We also found that distance to school was the strongest factor influencing ACS in both generations, with longer distances reducing the likelihood of active commuting. Family income was positively associated with ACS among G1 only.

Previous studies have reported positive associations between parents’ current commuting behaviours and their children’s ACS [22, 27, 35]. Parental modelling of active commuting has been positively linked with ACS in adolescents [22]. Other studies suggest that children are more likely to commute actively if their parents value PA [14, 24], hold favourable beliefs about ACS [27], or appreciate social interactions during the commute [36]. However, research on the intergenerational continuity of ACS remains limited. Our findings align with those of Ziviani *et al.* [14], who identified parents’ histories of ACS as a significant predictor of their children’s engagement in ACS. The link between parental and offspring ACS may arise from parental behaviours and attitudes formed during their own youth, which can influence family norms and their children’s lifestyle choices. Parents with experiences of ACS from their youth may be more inclined to encourage similar commuting habits in their children, thereby passing down active commuting behaviours across generations. Conversely, parents who did not actively commute to school may be less likely to prioritize or encourage ACS for their children. Parents can enhance their children’s participation in PA or ACS by providing instrumental support such as bikes, walking, or cycling to school with their children, establishing or eliminating barriers to ACS, and positively reinforcing their children’s participation in ACS [10, 18, 20, 37].

Additional analyses by school grades indicated that the transmission of ACS from parents to offspring is most pronounced in primary school. This aligns with findings that intergenerational transmission is age-dependent, with parental influence on children decreasing as they grow older [15]. Parental examples, attitudes, and actions have the greatest influence at younger ages, likely due to children’s reliance on caregivers and parental control. In contrast, during adolescence, parental influence decreases while the impact of friends and social norms increases [18, 37, 38]. In our study, the positive association between parental and offspring ACS was the weakest and statistically not significant among lower secondary school-aged (13–15 years) participants. Among upper secondary school-aged, only a trend towards a positive association between parental and offspring ACS was found. However, it should be noted that the results by school grade should be viewed with caution, as the model fit indices showed partly inadequate fit. Moreover, the number of participants was especially low among lower and upper secondary school-aged groups, potentially limiting the statistical power of the analyses.

Comparable to previous studies on the intergenerational transmission of PA [9, 39], we observed only modest positive associations between parental and offspring ACS. ACS is influenced by multiple factors beyond family-related ones, with environmental factors, particularly distance to school, being the most important and consistent across different studies [8]. In our study, distance to school was the strongest determinant of ACS in both generations, with the prevalence of ACS decreasing sharply as home–school distances increased. As expected, home–school distances were generally longer, and the proportions of ACS were lower in higher school grades in both generations. Shorter home–school distances at the primary school level could also partly explain the higher transmission of ACS at this age compared to higher school grades. Parents may support ACS by prioritizing supportive environments for ACS, especially at primary school, such as accessible home-school distances and safe routes to school. As reported earlier [28], family income was not associated with ACS among G2.

ACS was more common among the older generation (parents) than the younger generation (offspring), aligning with previous research showing that the levels of ACS have decreased in recent decades in various countries [8], including Finland [6, 7]. Intergenerational differences in ACS favouring the older generation were most pronounced among upper secondary school-aged participants. However, no notable differences were observed in home–school distances between the generations, which could have explained the lower ACS in the younger generation. In general, the levels of ACS were relatively high, exceeding 60% for both generations in the full sample. With shorter distances, the proportions of ACS were much higher, similar to those reported by Kallio *et al.* [40], who found over 80% prevalence of ACS among Finnish students aged 10–16 during the summer season for home–school distances of less than 5 km. The relatively high levels of ACS in Finland and other Nordic countries are likely due to a societal emphasis on ACS, safe urban planning, and relatively well-established infrastructure for walking or cycling. These conditions can also contribute to the transmission of ACS from one generation to the next.

This study has limitations. Firstly, the sample size for analyses by school grade was relatively small, and the fit indices for these models were partly inadequate, suggesting that these results should be interpreted with caution. The sample size was also insufficient for gender-specific analyses, which is important as intergenerational transmission of PA may differ between parents and children of different genders [15]. In the present study, data were available from only one parent (either mother or father) originally recruited in 1980, resulting in parent–offspring pairs of various gender combinations. However, the results were adjusted for gender for both generations. Furthermore, there were siblings in the G2 sample, and one parent could be included in several parent–offspring pairs of different ages. However, family clustering of the data was accounted for in the analyses. Finally, the age scales for G1 and G2 differed slightly, which may have impacted the findings.

The strengths of this study include a unique prospective design with almost 700 parent–offspring pairs of similar ages. ACS information was collected from different school grades, and several important covariates of ACS from both generations and factors affecting intergenerational transmission were considered. The seasonal variation in ACS, typical for Nordic countries, was also taken into account, as separate questions were available for summer and winter commuting. Similar questions were applied to both generations for accessing ACS and its covariates.

## Conclusions

In conclusion, we found a modest positive link between parents’ ACS during their youth and their offspring’s ACS at similar ages, after adjusting for several key covariates. This intergenerational association highlights the role of parental behaviour and attitudes in shaping their children’s commuting habits. Ensuring accessible distances to school also remains essential, given its strong effect on ACS. Promoting ACS could have a lasting impact on future generations, fostering sustainable and healthy commuting behaviours. Further research is needed to explore factors influencing this link and to develop specific strategies to promote ACS among youth. Ultimately, fostering a culture of active commuting can improve PA and overall health for both current and future generations.

## Supplementary Material

ckaf084_Supplementary_Data

## Data Availability

The data underlying this article cannot be shared publicly due to the local legal restrictions concerning the distribution of all personal information. The data will be shared on reasonable request to the YFS Data Sharing Committee (youngfinnsstudy.utu.fi). Key points In this study, 660 parent–offspring pairs in Finland reported their school commuting habits, parents during 1980–86, and offspring in 2018. Parents’ ACS during their youth was positively, albeit modestly, associated with their children’s ACS at similar ages, suggesting that parental behaviours and attitudes formed during their own youth can influence their children’s commuting habits. Distance to school was the strongest determinant of ACS in both generations, highlighting the need for schools to be located within accessible distances for the students.
