# Supplementary material for: Continuity of active commuting to school across two generations: the Cardiovascular Risk in Young Finns Study
Source: Eur J Public Health. 2025 Jun 3;35(4):745–51. doi: 10.1093/eurpub/ckaf084 (PMC12311363; doi:10.1093/eurpub/ckaf084)
Supplement: ckaf084_Supplementary_Data [file ckaf084_supplementary_data.docx]

**Supplementary Table S1**. Sample structure showing the number of parent-offspring pairs with valid data on the covariates of ACS across different school grades and ages within school grades.

|  | **G1 Age** | | | |  | **School grade** | | |
| --- | --- | --- | --- | --- | --- | --- | --- | --- |
| **G2 Age** | **9** | **12** | **15** | **18** |  | **Primary** | **Lower Secondary** | **Upper Secondary** |
| **7**  **8**  **9**  **10**  **11**  **12** | 36 | 4 |  |  |  | 40 |  |  |
|  | 44 | 4 |  |  |  | 48 |  |  |
|  | 69 | 5 |  |  |  | 74 |  |  |
|  | 61 | 5 |  |  |  | 66 |  |  |
|  | 24 | 54 |  |  |  | 78 |  |  |
|  | 23 | 52 |  |  |  | 75 |  |  |
| **13**  **14**  **15** |  |  | 46 |  |  |  | 46 |  |
|  |  |  | 52 |  |  |  | 52 |  |
|  |  |  | 57 |  |  |  | 57 |  |
| **16**  **17**  **18**  **19**  **20** |  |  |  | 28 |  |  |  | 28 |
|  |  |  |  | 43 |  |  |  | 43 |
|  |  |  |  | 17 |  |  |  | 17 |
|  |  |  |  | 15 |  |  |  | 15 |
|  |  |  |  | 21 |  |  |  | 21 |
|  |  |  |  |  |  | 381 | 155 | 124 |

Note. The data of G1 participants (the parents) were matched with the G2 participants (the offspring) by school grade and age. For the 7‒10-year-old offspring at primary school, parental data was obtained from the follow-up when the parent was 9 years old. For the 11‒12-year-old offspring at primary school, the parents' follow-up data at age 12 was used. If these specific parental data were unavailable, data for the 7-10-year-old offspring were taken from the follow-up when the parents were 12 years old, and for the 11-12-year-old offspring, data were taken from the follow-up when the parents were 9 years old, if available. For the 13-15-year-old offspring at lower secondary school, the parental data was collected from the follow-up at age 15. Correspondingly, for the 16-20-year-old offspring at upper secondary school, the parental data was derived from the follow-up of age 18.

**Supplementary Table S2.** Characteristics of participants by generation and school grade.

|  | **Primary (n = 381)** | |  | **Lower Secondary (n = 155)** | |  | **Upper Secondary (n = 124)** | |
| --- | --- | --- | --- | --- | --- | --- | --- | --- |
|  | **G1 generation, parents** | **G2 generation, offspring** |  | **G1 generation, parents** | **G2 generation, offspring** |  | **G1 generation, parents** | **G2 generation, offspring** |
| Gender, n (%) |  |  |  |  |  |  |  |  |
| Female | 199 (52.2) | 189 (49.6) |  | 82 (52.9) | 76 (49.0) |  | 70 (56.5) | 77 (62.1) |
| Male | 182 (47.8) | 192 (50.4) |  | 73 (47.1) | 79 (51.0) |  | 54 (43.5) | 47 (37.9) |
| Age, years (mean, SD) | 10.0 (1.4) | 9.8 (1.6) |  | 15.0 (0) | 14.0 (0.8) |  | 18.0 (0) | 17.7 (1.4) |
| Distance to school, km (mean, SD)^a^ | 2.3 (2.4) | 2.5 (2.7) |  | 4.0 (3.6) | 4.3 (3.6) |  | 5.4 (3.7) | 5.4 (3.6) |
| Living area^b^, % |  |  |  |  |  |  |  |  |
| Rural | 60.1 | 31.0 |  | 48.5 | 32.9 |  | 39.5 | 26.6 |
| Urban | 39.9 | 69.0 |  | 54.2 | 67.1 |  | 60.5 | 73.4 |
| Parental education^b^, % |  |  |  |  |  |  |  |  |
| Elementary | 42.3 | 4.5 |  | 55.5 | 1.9 |  | 60.5 | 7.3 |
| Secondary | 40.7 | 32.5 |  | 29.7 | 41.3 |  | 30.6 | 44.4 |
| Academic | 17.1 | 63.0 |  | 14.8 | 56.8 |  | 8.9 | 48.4 |
| Family income^b^, % |  |  |  |  |  |  |  |  |
| Low | 20.7 | 18.9 |  | 21.9 | 24.5 |  | 19.4 | 24.2 |
| Middle | 38.8 | 42.3 |  | 31.6 | 32.9 |  | 29.8 | 37.1 |
| High | 40.4 | 38.8 |  | 46.5 | 42.6 |  | 50.8 | 38.7 |
| Commuting mode, % |  |  |  |  |  |  |  |  |
| Passive | 21.8 | 24.8 |  | 40.0 | 43.2 |  | 48.8 | 63.7 |
| Active | 78.2 | 75.2 |  | 60.0 | 56.8 |  | 51.2 | 36.3 |

Values are percentages unless otherwise noted. ^a^ Truncated at 10.0 km, ^b^ Missing data are supplemented with data from previous follow-ups.

**Supplementary Table S3.** Estimated probabilities and odds ratios of ACS in the G2 generation by home-school distances of 1-10 km and parental ACS categories.

|  | **Estimated probability of ACS** | | **Odds ratio for ACS** | |
| --- | --- | --- | --- | --- |
| **Distance to school (km)** | **Not ACS parent^a^** | **ACS parent^b^** | **ACS vs. not ACS parent^c^** | **1 km addition^d^** |
| 1 | 94.3 % | 95.6 % | 1.31 | 0.42 |
| 2 | 87.7 % | 90.1 % | 1.28 | 0.46 |
| 3 | 77.0 % | 80.8 % | 1.26 | 0.49 |
| 4 | 62.6 % | 67.4 % | 1.24 | 0.51 |
| 5 | 46.1 % | 51.3 % | 1.23 | 0.51 |
| 6 | 30.3 % | 34.9 % | 1.23 | 0.50 |
| 7 | 17.5 % | 21.0 % | 1.25 | 0.46 |
| 8 | 8.8 % | 11.0 % | 1.28 | 0.43 |
| 9 | 3.8 % | 5.0 % | 1.33 | 0.39 |
| 10 | 1.4 % | 2.0 % | 1.44 |  |

^a^ Offspring with a passively commuted parent, ^b^ Offspring with an actively commuted parent, ^c^ Actively commuted parent versus passively commuted parent, ^d^ One kilometre longer distance for a participant with an actively commuted parent.

Note. In the transformation of the path coefficients into a probability scale, values for all variables in the model must be assumed. In this additional analysis, home-school distances and parental ACS are varied, and all other covariates are fixed at their mean or mode levels. The linear predictor obtained from the path model regarding ACS in G2 generation was (inverse probit) transformed into probabilities, which were subsequently compared by calculating odds ratios.

**
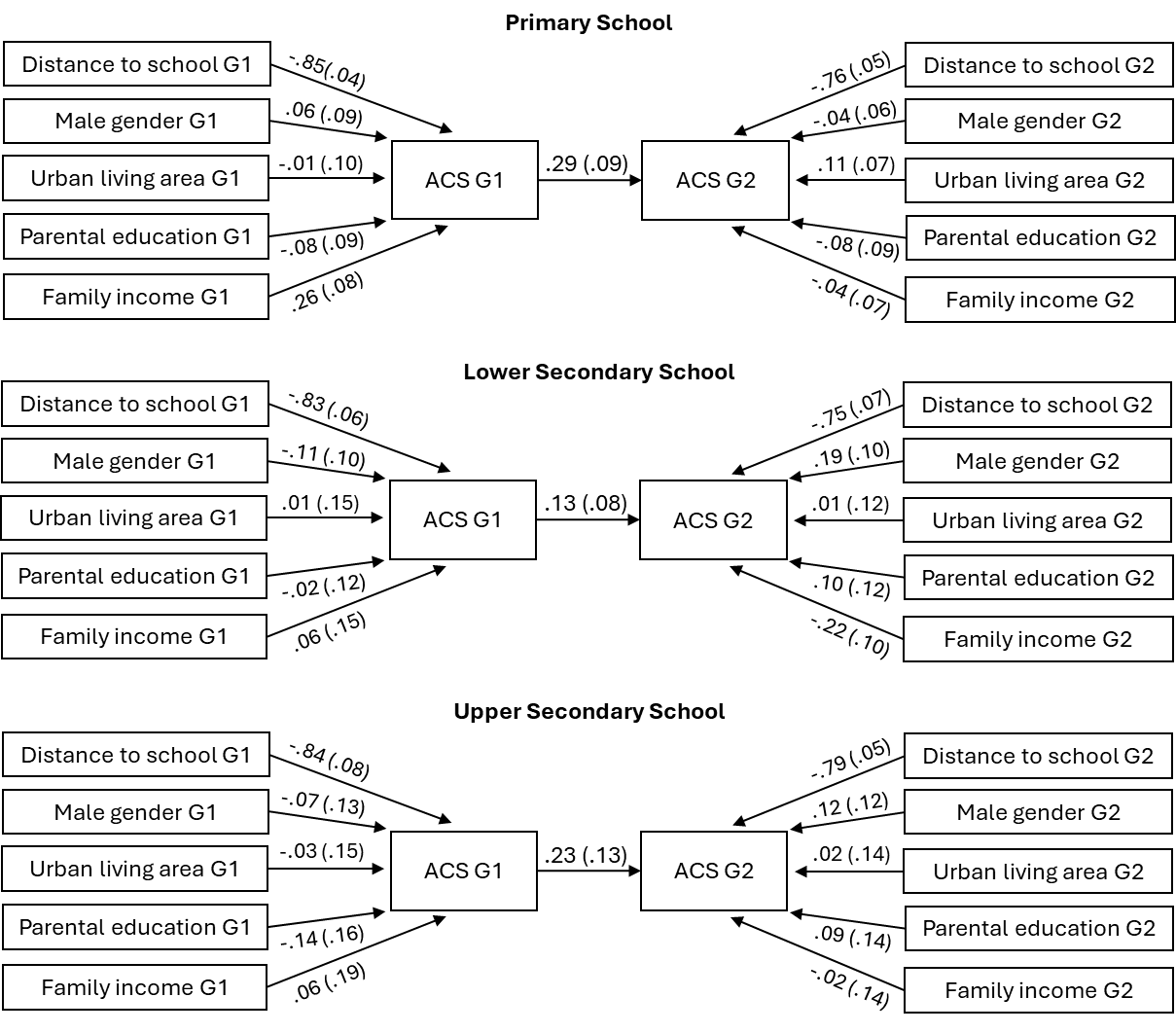
**

**Supplementary Figure S4**. Directed acyclic graph of the path model of ACS across two generations with its covariates, separately for the school grades (primary school n = 381, lower secondary school n = 155, and upper secondary school n = 124, for both G1 and G2). Values are standardized regression coefficients (SE). Model fit: primary school: X^2^ = 26.4 (df 11), p = 0.006; CFI = 0.892, TLI = 0.774, RMSEA = 0.061, and SRMR = 0.144; lower secondary school: X^2^ = 9.86 (df 11), p = 0.543; CFI = 1, TLI = 1, RMSEA = 0, and SRMR = 0.140; upper secondary school: X^2^ = 6.16 (df 11), p = 0.863; CFI = 1, TLI = 1, RMSEA = 0, and SRMR = 0.148).
